# Prevalence of Syphilis among Pregnant Women Attending Antenatal Care Clinic, Sede Muja District, South Gondar, Northwest Ethiopia

**DOI:** 10.1155/2019/1584527

**Published:** 2019-07-14

**Authors:** Getachew Yideg Yitbarek, Belete Achamyelew Ayele

**Affiliations:** ^1^Department of Biomedical Sciences (Medical Physiology Unit), College of Health Sciences, Debre Tabor University, Debre Tabor, Ethiopia; ^2^Department of Epidemiology, Sede Muja Woreda Health Office, South Gondar Zone, Ethiopia

## Abstract

**Background:**

Syphilis is a disease caused by bacteria called* Treponema pallidum*. Major rout of transmission of this bacterium was through sexual and contact with mucocutaneous lesion. Untreated syphilis during pregnancy can greatly affect pregnancy outcome, resulting in spontaneous abortion and stillbirth.

**Objective:**

The objective of the study was to determine the seroprevalence of syphilis and associated factors among pregnant women attending antenatal care unit at Sede Muja district, Northern Ethiopia.

**Methods and Materials:**

Facility based cross-sectional study was conducted from November 2018 to January 2019 in two health centers from Sede Muja district, Northwest Ethiopia. The study included a total of 210 participants. The entire participants were recruited by systematic random sampling method after proportional allocation of the sample size in the two health centers. Sociodemographic and clinical data were collected by semistructured questioner. Two milliliter of blood was collected to determine seroprevalence of syphilis using VDRL test method. The data was analyzed by using SPSS version 21. The association between independent and dependent variables was determined using chi-square. P-value less than 0.05 was considered as statistically significant.

**Result:**

Seroprevalence of syphilis was found to be 1.9% (95 CI, 0.5-3.5%). Women with multiple sexual partners and late trimester of pregnancy during first ANC visit were found to be significantly associated with seropositive syphilis.

**Conclusion:**

Syphilis remains a major problem in the study area. Seroprevalence of syphilis was significantly associated with women who have multiple sexual partners and late first ANC visit attendees in the study area. Therefore it is recommended to give health education about syphilis etiology and transmission as well as creating awareness about the importance of early ANC visit and follow-up regarding syphilis prevention.

## 1. Introduction

Syphilis is a systemic disease caused by* Treponema pallidum *which can be spread by sexual contact, blood transfusion, and vertical transmission which is a classical example of a disease that can be successfully controlled by effective public health measures due to the availability of a sound diagnostic test and effective and economical treatment options. The World Health Organization (WHO) estimates that 10-12 million new infections of syphilis occur every year. Infection rates show extreme variation between countries of the same region as well as various subcategories of populations studied with syphilis 2009 [1]. Seroprevalence during pregnancy is generally low in developed countries: it ranges from 0.02% in Europe to 4.5% in parts of the United States [2]. In contrast, there has been a dramatic increase in the incidence of congenital syphilis in rural areas of Eastern Europe and Central Asia. High rates of syphilis seropositivity have consistently been reported at antenatal clinics in Africa (3–18%) [3]. In Zambia, 24% of all stillbirth could be attributed to syphilis and congenital syphilis was implicated in 30% of all perinatal mortality [4]. A study conducted in Debre Berhan town, Ethiopia, showed that the prevalence of syphilis was 5.1% [5].

The mother can transmit the infection transplacentally to the fetus or during passage through the birth canal. Until recently, a commonly held but erroneous obstetric principle stated that infection of the fetus does not occur before 18 weeks. But Silver and immunofluorescence staining of the fetal tissue or polymerase chain reaction and rabbit infectivity testing of amniotic fluid showed that T.* pallidum *gains access to the fetal compartment as early as 9-10 weeks [6].

Appropriate treatment of pregnant women often prevents such complication. The main problem is inability to identify the infected women and get them to treatment. Screening in the first trimester with VDRL and RPR test combined with confirmation of reactive individuals with treponemal tests such as fluorescent treponemal antibody absorption (FTA-Abs) assay is a cost effective strategy.

Syphilis in pregnant women can result in adverse outcomes of pregnancy in up to 80% of cases, such as stillbirth and spontaneous abortion (40%), perinatal death (20%), and serious neonatal infections and low-birth weight babies (20%). Syphilis has also acquired a new potential for morbidity and mortality through association with increased risk for HIV [7].

Prevalence rate of syphilis infections among pregnant mothers differs between countries and regions depending on a number of factors such as the national HIV prevalence and culture of the general population. Even if there are limited published studies in different places of Ethiopia, no studies have been conducted at particular places under study; as a result there is scarcity of information about the true burden and determinant factors of syphilis infection in the study area (Sede Muja district).

## 2. Materials and Methods

The study was conducted in two health centers found in Sede Muja district (Muja health center and Sede Adada health center), south Gondar Zone of Amhara Region, about 786 kilometers from northern part of Addis Ababa. There are 3 health centers and 10 health posts in the district which are currently on service. Facility based cross-sectional study was conducted to assess seroprevalence of syphilis and associated factors among pregnant women attending antenatal care in Sede Muja district from November 2018 to January 2019.

### 2.1. Sample Size Determination

Sample size was statistically calculated based on single population proportion formula by taking 5.1% prevalence of syphilis infection from previous study [5] and desired precision of 3%. A 95% confidence level and 5% contingency were considered. Finally, a total of 217 study participants were included.

### 2.2. Sampling Technique and Procedure

Two health centers were selected in the district that gives antenatal care services in the district. A total of 1860 pregnant women were registered at the antenatal care clinics at all public health institutions in the district since February 2018 and January 2019. Samples proportional to pregnant women in the selected health facilities were allocated. Lists of pregnant women were prepared using unique identification numbers from records found in ANC clinics and sampled by systematic random sampling using the registers sampling frame. To select the first eligible study participant, a simple random sampling method was used and the study units were selected until the required sample size was achieved. All pregnant women who had ANC records with complete information were included in the study while all pregnant women who had ANC records with incomplete information were excluded.

### 2.3. Data Collection Method

Data was collected by trained nurses from record log book and patient charts using semistructured questionnaires. The tool was pretested on the ANC chart on another health center which provides ANC service and which is not part of the study and necessary adjustments were made in the data collection instrument. Regarding syphilis test two milliliter of blood was drawn from the mother and VDRL test was done.

### 2.4. Data Processing and Analysis

The collected data was clearly summarized, filled, and analyzed by using SPSS version 21. Descriptive statistics was employed and the result was presented by using Table 1. The difference in mean age of the participant acording to their serostatus for syphilis was compared using Independent samples Student* t*-test. The association between independent and dependent variables was determined using chi-square. P-value less than 0.05 was considered as statistically significant.

### 2.5. Ethical Consideration

Ethical approval was obtained from Sede Muja woreda administration and official permission was obtained from Sede Muja woreda health office and from public health institutions where the actual data were collected. For all study participants the objective of the study was explained and written informed consent was obtained.

## 3. Result

A total of 210 pregnant women were enrolled to the study making a response rate of 97.2%. All of them were consented, interviewed, and provided clinical samples. From the women tested for syphilis, four women were found seropositive for syphilis. The prevalence of syphilis in this study was 1.9% (95 % CI, 0.5-3.8).

From total of participants, majority (73.8%) of them were found in age group of 21-30 years old. 74.8 % of the participants were married. 60.5 % of them were from urban while other 39.5% were from rural area. 45.3 % of them had monthly income of less than 1000 Ethiopian birr. Regarding occupation of the participants 38.6 % of them were housewives while 29.5 % were merchants. 93.8% have no multiple sexual partners while other 6.2% had multiple sexual partners. The age, marital status, monthly income, residency, and occupation of the participant showed no significant association while trimester of pregnancy and multiple sexual status showed significant association with the acquirement of syphilis.

**Figure 1:**
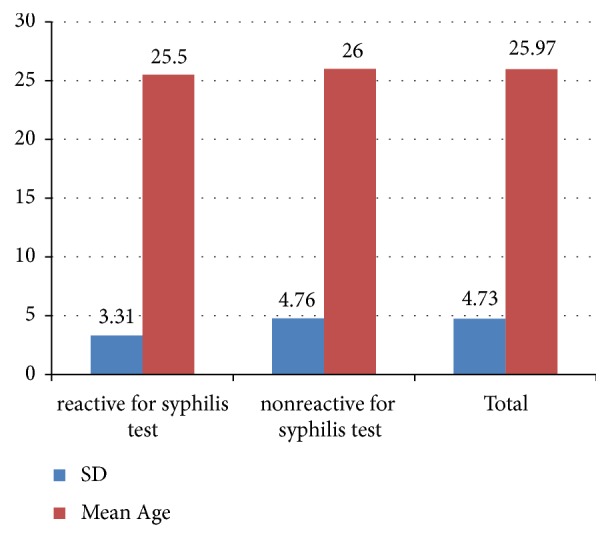
Age difference in pregnant mothers by their syphilis serostatus. Independent samples Student's* t*-test: t (df = 208) = 3.245, p = 0.841.

Mean ages of pregnant mothers who are seropositive for syphilis were 25.5 whereas mean ages of pregnant mothers who are seronegative for syphilis were 26. The difference in mean ages of pregnant mothers according to their serostatus for syphilis was not statistically significant, p = 0.841 (Figure 1).

## 4. Discussion

Syphilis remains a major cause of morbidity and mortality in the world despite the availability of effective treatment. The seroprevalence of syphilis in this study was 1.9% (95 % CI, 0.5-3.5) which is comparable to the studies conducted in Jimma (1.1%), Debre Berhan (1.8%), and Addis Ababa (2.9%) [5, 8, 9]. The seroprevalence of syphilis in this study is also comparable with other studies done in Nigeria (2.2%) [10] and brazil ( 1.02%) [11] but lower than a study done in Zambia (8.2%) [12] and Uganda (5.1%) [13]. The observed differences might be due to differences in geographic sites and time-period, sociocultural and economic factors, and differential access to syphilis diagnosis and treatment.

This study also showed smaller seroprevalence of syphilis among pregnant mothers from study done in Wolaita Sodo University (3.7%) and northern Ethiopia (13.7%) [5]. This may be due to the difference in sexual behavior and practices, climatic conditions, type of population being studied, sociodemographic characteristics of the study populations, and sample size difference. However, the prevalence syphilis was found higher than a study done in Pakistan (0.9%) [8]. The possible reason for the difference with study done in Pakistan might be due to the difference in syphilis diagnostic test used.

The prevalence of syphilis in this study was higher in rural (3.1%) than urban (0.9%) areas which goes in line with the national ANC sentinel survey of Ethiopia in 2009 (6.7%) in rural and (4.7%) from urban setting [5]. Similarly, according to the national ANC sentinel survey of Ethiopia, in 2012, the syphilis prevalence rounds were 0.7% in urban and 1% in rural sites [14]. It could be due to lack of awareness, poor knowledge, and poor understanding of health service at the ANC in rural pregnant women which help in adequate prevention of sexually transmitted diseases. Urban residents might have better health seeking behavior to get tested and treated earlier which could alter the status of syphilis.

In this study late trimester of pregnancy has been found to be associated with seropositive syphilis which is in line with study done in Yirga Alem, Ethiopia [15]. High seroprevalence of syphilis in those women who visited ANC services more than once and women at third trimester suggests that those women who visited the clinic more than once may not properly either screened or treated in their first ANC visit. According to WHO, delayed antenatal care (after the 12th gestational week) is one of the barriers for the control of adverse pregnancy outcomes which is an indicative that many pregnant women that have been diagnosed and treated after the recommended gestational age are more likely to attain poor outcomes of pregnancy.

The present study showed that 93.8% of the participants had no multiple sexual partners while and 6.2 % were having multiple sexual partners. Among those who had multiple sexual partners 30 % of them were positive for syphilis partner. The prevalence of syphilis in individuals having multiple sexual partners is still higher and there is a significant association which is in line with a study done in Jimma, Ethiopia [8]. On the other hand, this study revealed that all of the participants who are positive for syphilis were found in the age range between 26 and 35 years. This might be attributable to the fact that sexual activity will be higher in this age range where it is a risk for having multiple sexual partner and further for syphilis.

## 5. Conclusion

The prevalence of syphilis in this study was 1.9 % (95% CI, 0.5-3.5). Syphilis seropositive cases were observed among married women who were housewives by occupation and those who come from rural areas. Multiple sexual partners and first ANC visit in late trimester pregnancy were found to be a risk factor for syphilis. Although VDRL rather than TPHA was used for syphilis test (one of the limitation of the study), this study showed that syphilis still remained one of the significant public health problems in our study area. Therefore, there should be health education about syphilis, its risk factors and the importance of ANC follow-up in early pregnancy. Furthermore, associated factors for syphilis transmission need to be further studied in large sample size including study in the general population to raise awareness of mothers.

## Figures and Tables

**Table 1:** Sociodemographic characteristics and syphilis serology status of pregnant women attending antenatal care clinic in Sede Muja district, South Gondar, Ethiopia, 2019.

Variable		RPR result								
		Positive (n=4)		Negative (n=206)		Total (n=210)				
		No	%	No	%	No	%	x^2^	Df	P value
Age	15-20	0	0	22	10.7	22	10.5	4.24	4	P=0.375
	21-25	0	0	78	37.9	78	37.1			
	26-30	3	75	74	35.9	77	36.7			
	31-35	1	25	25	12.1	26	12.4			
	>36	0	0	7	3.4	7	3.3			

Marital status	Single	0	0	14	6.8	14	6.7	2.34	3	p=0.51
	Married	3	75	154	74.8	157	74.8			
	Widowed	0	0	23	11.2	23	11			
	Divorced	1	25	15	7.3	16	7.6			

Residency	Urban	1	25	126	61.2	127	60.5	2.15	1	p=0.14
	Rural	3	75	80	38.8	83	39.5			

Monthly income	<1000	2	1.1	80	44.2	82	45.3	0.25	3	P=0.13
	1000-2000	0	0	59	32.6	59	32.6			
	≥2000	0	0	40	22.1	40	22.1			

Occupation	Housewife	3	75	78	37.9	81	38.6	2.9	3	2.9
	Government employee	1	25	50	24.3	51	24.3			

	Student	0	0	16	7.8	16	7.6			
	Merchant	0	0	62	30.1	62	29.5			

Multiple sexual partners	Yes	4	100	9	4.4	13	6.2	61.8	1	0.000
	No	0	0	197	95.6	197	93.8			

Trimester of pregnancy during 1^st^ ANC visit	First	0	0	135	65.5	135	64.3	8.31	2	0.016
	Second	2	50	46	22.3	48	22.9			
	Third	2	50	25	12.1	27	12.9			

Total		4	1.9	206	98.1	210	100			

## Data Availability

The data used to support the findings of this study are available from the corresponding author upon request.

## Additional Points

*Study Limitations.* Nevertheless, sample size limited our ability to show significance for many demographic variables and the study could not address the strength of the association of factors with the outcome. We did not evaluate birth outcomes of mothers treated for syphilis due to the cross-sectional nature of the study. Regardless of the depicted limitations, this study ultimately adds supportive information on increased prevalence of syphilis in the study area.
